# Bojungikgi-tang improves skin barrier function and immune response in atopic dermatitis mice fed a low aryl hydrocarbon receptor ligand diet

**DOI:** 10.1186/s13020-023-00806-9

**Published:** 2023-08-13

**Authors:** You Mee Ahn, Sarah Shin, Ji-hye Jang, Jeeyoun Jung

**Affiliations:** KM Science Research Division, Korea Institute of Oriental Medicine, Daejeon, 34054 Republic of Korea

**Keywords:** BJIKT, Low-AhR ligand diet, Atopic dermatitis, Skin barrier, Inflammation, Immune response

## Abstract

**Background:**

The aryl hydrocarbon receptor (AhR) is a transcription factor that plays a crucial role in regulating the immune system and maintaining skin barrier function. AhR signaling is pivotal in the pathogenesis of inflammatory diseases such as atopic dermatitis (AD), and the absence of AhR ligands further contributes to the progression or worsening of AD symptoms.

**Methods:**

AD was induced with 2,4-dinitrochlorobenzene (DNCB), and Bojungikgi-tang (BJIKT) was administered orally daily for 10 weeks. Serum IgE, splenocyte IL-4, and IFN-γ levels, skin barrier genes, and AhR target gene expressions were analyzed using RNA-sequencing analysis. Spleen tissues were extracted for fluorescence-activated cell sorting (FACS) analysis to analyze the effect of BJIKT on immune responses. A correlation analysis was conducted to analyze the correlation between immune markers and skin barrier genes and AhR target genes.

**Results:**

BJIKT effectively improved AD symptoms in AD mice fed a low AhR ligand diet by reducing neutrophil and eosinophil counts, lowering IgE levels in the blood, and decreasing IL-4 and IFN-γ levels in the splenocytes. Additionally, BJIKT significantly reduced epithelial skin thickness and transepidermal water loss (TEWL) values and reversed the decreased expression of skin barrier genes. BJIKT also considerably altered the expression of AhR target genes, including Ahr, Ahrr, cytochrome P450 1A1 (CYP1A1), and CYP1B1. Furthermore, AhR target pathway genes were negatively correlated with immune cell subtypes, including CD4 + and CD8 + T cells and macrophages (CD11b + F4/80 +) at the systemic level.

**Conclusions:**

BJIKT can regulate AhR activation and may help reduce inflammation in AD by regulating the expression of skin barrier genes and immune responses.

## Background

The aryl hydrocarbon receptor (AhR) signaling pathway plays a critical role in various physiological processes, including immune responses and skin homeostasis [1]. Activation of AhR, a ligand-dependent transcription factor, triggers a cascade of signaling events, regulating gene expression and cellular functions.

In the skin, AhR activation is vital for maintaining a healthy skin barrier under normal physiological conditions. It controls the expression of key proteins involved in the formation and maintenance of the epidermal barrier, such as filaggrin, involucrin, and loricrin [2–4]. These proteins are essential for the proper formation of the outer keratinized layer of the skin. AhR activation also induces the expression of xenobiotic metabolizing enzymes, including cytochrome P450 (CYP) 1A1 (CYP1A1) and CYP1B1, which contribute to the regulation of barrier function by metabolizing AhR ligands [5]. Moreover, AhR signaling has been demonstrated to suppress the production of pro-inflammatory cytokines, inhibit the abnormal proliferation of keratinocytes, and enhance the expression of proteins crucial for maintaining the integrity and function of the epidermal barrier [6]. Dysregulation or absence of AhR ligands can disrupt these processes, leading to immune dysregulation, increased inflammation, and compromised skin barrier function observed in atopic dermatitis (AD) [7, 8].

Tapinarof, an AhR agonist, has been approved for the treatment of plaque psoriasis and atopic dermatitis [9]. Additionally, compounds such as 6-formylindolo [3,2-b] carbazole (FICZ) and indole-3-aldehyde (IAld) have demonstrated effectiveness in AD treatment via the activation of the AhR-ARNT-FLG axis [10, 11]. Although Bojungikgi-tang (BJIKT), an oriental medicine, has been reported to be effective in treating immune diseases, such as atopy, allergy, and asthma [12–15], its specific mechanism of action on the skin barrier, AhR target signaling, and immune response in AD mice fed a low AhR ligand diet remains poorly understood.

Therefore, our study aimed to investigate the impact of a low AhR ligand diet on impaired skin barrier function and immune regulation in AD mice. Furthermore, we sought to elucidate the potential therapeutic effects and underlying mechanisms of BJIKT. Through a comprehensive analysis of these aspects, our study aimed to provide valuable insights into the effects of BJIKT on the skin barrier, modulation of AhR signaling, and immune response in AD mice subjected to a low AhR ligand diet. This research enhances our comprehension of novel therapeutic strategies for AD and highlights the promising potential of BJIKT as a treatment option.

## Methods

### Preparation of BJIKT water extract and ultra-high performance liquid chromatography-quadrupole time-of-flight mass spectrometry (UHPLC/Q-TOF–MS) analysis

The herbal formula BJIKT is composed of eight herbal medicines: astragalus root (300 g), atractylodes rhizome white (200 g), ginseng (200 g), licorice (200 g), angelica gigas root (100 g), citrus unshiu peel (100 g), bupleurum root (60 g), and cimicifuga rhizome (60 g). The dried herbs, as mentioned earlier, were chopped and mixed, then extracted with distilled water using a crude extraction system (95 ± 5 °C, 3 h). The insoluble particles were removed by passing the extract through a 53 μm mesh strainer. The resulting filtrates were concentrated and evaporated until dry using a freeze-dryer. The final dried BJIKT extracts (0.38 kg, 31.06% yield) were homogenized and stored in an airtight container.

A qualitative analysis of BJIKT was performed using UHPLC (1290 infinity II LC system, Agilent Technologies, Santa Clara, CA, USA) combined with a Q-TOF MS (6546 Q-TOF, Agilent Technologies) system to identify its chemical composition. The dried powder of BJIKT was dissolved in a 50% MeOH solution at a concentration of 0.1 mg/mL. The recovered signal was separated using a Zorbax Extend-C18 column (80 Å, 2.1 × 50 mm, 1.8 µm, Agilent Technologies) on a binary gradient system. The mobile phase consisted of water containing 0.1% (v/v) formic acid (solvent A), and acetonitrile containing 0.1% (v/v) formic acid (solvent B). The flow rate was set to 0.1 mL/min for a total of 30 min of run time, and the injection volume of the BJIKT solution was 2 μL. The linear gradient of UHPLC was as follows: 5% B for 3 min, 5% to 50% B from 3 to 5 min, 50% to 85% B from 5 to 20 min, and then 85% B from 20 to 25 min, and then equilibration for 30 min. The mass spectrometer was operated in the positive ionization mode with a mass range of m/z 50–1700. The MS parameters were set as follows: Gas temperature, 325 °C; drying gas, 5 L/min; nebulizer, 15 psi; fragmentor, 125 V; skimmer, 65 V; and capillary voltage, 3500 V. A narrow isolation window (1.3 Da) was used to acquire the MS/MS data at a collision energy of 20 V. The acquired naked data were processed using MS-Dial software (Ver 4.80, http://prime.psc.riken.jp/), and the chemical constituents of BJIKT were identified based on the retention time, m/z of precursor, and the MS fragment pattern using all publicly available mass spectral databases obtained from RIKEN (http://prime.psc.riken.jp/ Metabolomics_Software/MS-DIAL/).

### Experimental animals

All animal procedures were approved by the Institutional Animal Care and Use Committee of the Korea Institute of Oriental Medicine (approval number: 20–050) and with the NIH Guide for the Care and Use of Laboratory Animals. Three-week-old male C57BL/6N mice were purchased from Saeron Bio Inc. (Gyeonggi-do, Republic of Korea). The mice had an adaptation period of one week in a specific pathogen-free animal facility at a constant room temperature (20–22 °C), humidity (40–60%), and a 12 h–12 h day and night cycle at the Korea Institute of Oriental Medicine. Following the adaptation period, the mice were either fed a normal chow diet or a low AhR ligand diet (ENVIGO [#TD130959]), and a low AhR ligand diet group was provided with AhR ligand-free diet paper bedding (#SSP0001, ALPHA-dri, Shepherd Specialty Papers, Watertown, TN, USA). Mice were fed with the low-AhR ligand diet for 12 weeks. After 2 weeks of a low AhR ligand diet, AD was induced with 2,4-dinitrochlorobenzene (DNCB), and BJIKT (1 g/kg/day in water) was administered by oral gavage daily at the same time. The specific dosage of BJIKT was determined by referring to existing literature, which provided valuable insights into the appropriate concentration for the experimental setup [16–18]. To induce AD, the dorsal skin of mice was treated twice a week with 1% DNCB mixed in a 3:1 acetone/olive oil solution.

### Analysis of neutrophils and eosinophils in mouse whole blood

Hematological analysis was performed on the blood samples collected in EDTA-treated tubes (BD Microtainer^®^ 365974; BD Biosciences, Franklin Lakes, NJ, USA) using capillary tubes (2501, Kimble Chase Life Science, Rockwood, TN USA). We utilized a specialized automated hematology analyzer, the HEMAVET^®^ 950FS (Drew Scientific, Miami Lakes, FL, USA), to measure the number of neutrophils and eosinophils in whole blood samples obtained through cardiac puncture. The HEMAVET^®^ 950FS is specifically designed for veterinary use and is capable of providing a complete blood count (CBC) analysis, including the enumeration of various blood cell types, such as neutrophils and eosinophils. This instrument employs diverse techniques, including impedance and optical measurements, to differentiate and quantify different cell types based on their size, shape, and staining properties. Following the analysis, the HEMAVET^®^ 950FS generates comprehensive results, including the counts of neutrophils and eosinophils [19, 20].

### Measurement of IgE

Blood samples were drawn from the mice, and the plasma was separated by centrifugation (1500×*g*, 20 min, 4 °C) and stored at − 80 °C. Plasma total IgE concentrations were measured using kits (Bethyl Laboratories Inc., Montgomery, TX, USA), according to the manufacturer's instructions.

### Measurement of transepidermal water loss (TEWL)

TEWL, the measurement of the quantity of water that passes from inside an animal's body to the surrounding atmosphere through the epidermal layer (skin) via diffusion and evaporation processes, was measured on the last day of the experiment (12 weeks). TEWL in mouse dorsal skin was measured under specific conditions of 20–22 °C and 45–55% humidity using a skin evaporative water recorder, the Tewameter TM300 (Courage + Khazaka electronic GmbH, Cologne, Germany). Measurements were recorded when the TEWL readings stabilized approximately 30 s after the probe was placed on the skin. Data were analyzed using a microprocessor and expressed in g/m^2^/h.

### Isolation of splenocytes and measurement of cytokines

Splenocytes were isolated as previously described [21]. After incubation for 24 h in a CO_2_ incubator, culture supernatants were assayed for cytokines. Interleukin (IL)-4 and interferon-gamma (IFN-γ) levels were analyzed using the Bio-Plex Pro Mouse Cytokine T helper (Th)1/Th2 Assay Kit (Bio-Rad Laboratories, Hercules, CA, USA).

### Flow cytometry analysis

After the isolation of splenocytes [21], the immune population was investigated using flow cytometry. Flow cytometry was performed as previously described [22]. Briefly, the cells were treated with RBC lysis buffer (BioLegend, San Diego, CA, USA), suspended in a cell-staining buffer (BioLegend), and non-specific staining was blocked using TruStain FcXTM PLUS. Subsequently, the cells were incubated for 30 min at 4  C in the dark with fluorescence-conjugated antibodies (1:100) obtained from BD Biosciences: anti-CD3, anti-CD4, anti-CD8, anti-CD69, anti-T-cell receptor (TCR) γδ, anti-CD11b, anti-F4/80, and anti-GR1. The cells were washed and examined using a BD LSRFortessa^™^ X-20 flow cytometer (BD Biosciences). All flow cytometric data acquired were analyzed with the FlowJo software (Tree Star, Ashland, OR, USA).

### Histological analysis

The dorsal skin tissues were fixed in a 10% neutral buffered formalin (10% NBF, HT501128; Merck, Darmstadt, Hessen, Germany) solution for 24 h. Tissues were fixed in paraffin-embedded Sects. (3–4 μm thick) and attached to slides. Histopathological changes were observed through hematoxylin and eosin (H and E) staining (H and E stain, ab245880; Abcam, Cambridge, UK) under light microscopy (EVOSTM M5000; Thermo Fisher Scientific, Waltham, MA, USA).

### RNA isolation and sequencing analysis

Total RNA was isolated from skin tissue using the TRIzol reagent (Invitrogen, Carlsbad, CA, USA). Only four skin tissue samples were randomly used for each group, and analysis was requested by EBIOGEN. RNA quality was assessed using an Agilent 2100 bioanalyzer with the RNA 6000 Nano Chip (Agilent Technologies), and RNA quantification was performed using an ND-2000 Spectrophotometer (Thermo Fisher Scientific). The cDNA library was constructed using the QuantSeq 3’ mRNA-Seq Library Prep Kit (Lexogen GmbH, Vienna, Austria) according to the manufacturer’s instructions. High-throughput sequencing was performed as single-end 75 sequencing using a NextSeq 500 (Illumina, Inc., San Diego, CA, USA).

Differential expression and ontologies of genes were identified using Excel-based Differentially Expressed Gene Analysis (ExDEGA) version 4.0.3 (EBIOGEN, Cambridge, MA, USA). Gene classification was based on searches performed using DAVID (http://david.abcc.ncifcrf.gov/) and Medline databases (http://www.ncbi.nlm.nih.gov/).

### Statistical analysis

All experimental data are presented as the mean ± standard error of the mean (SEM) and were analyzed using one-way analysis of variance (ANOVA) followed by Tukey's multiple comparison tests. Statistical significance between the groups was set at p < 0.05. Data visualization was performed using GraphPad Prism (version 9.0; GraphPad Prism Software, La Jolla, CA, USA).

## Results

### Chemical components in BJIKT

**Fig. 1:**
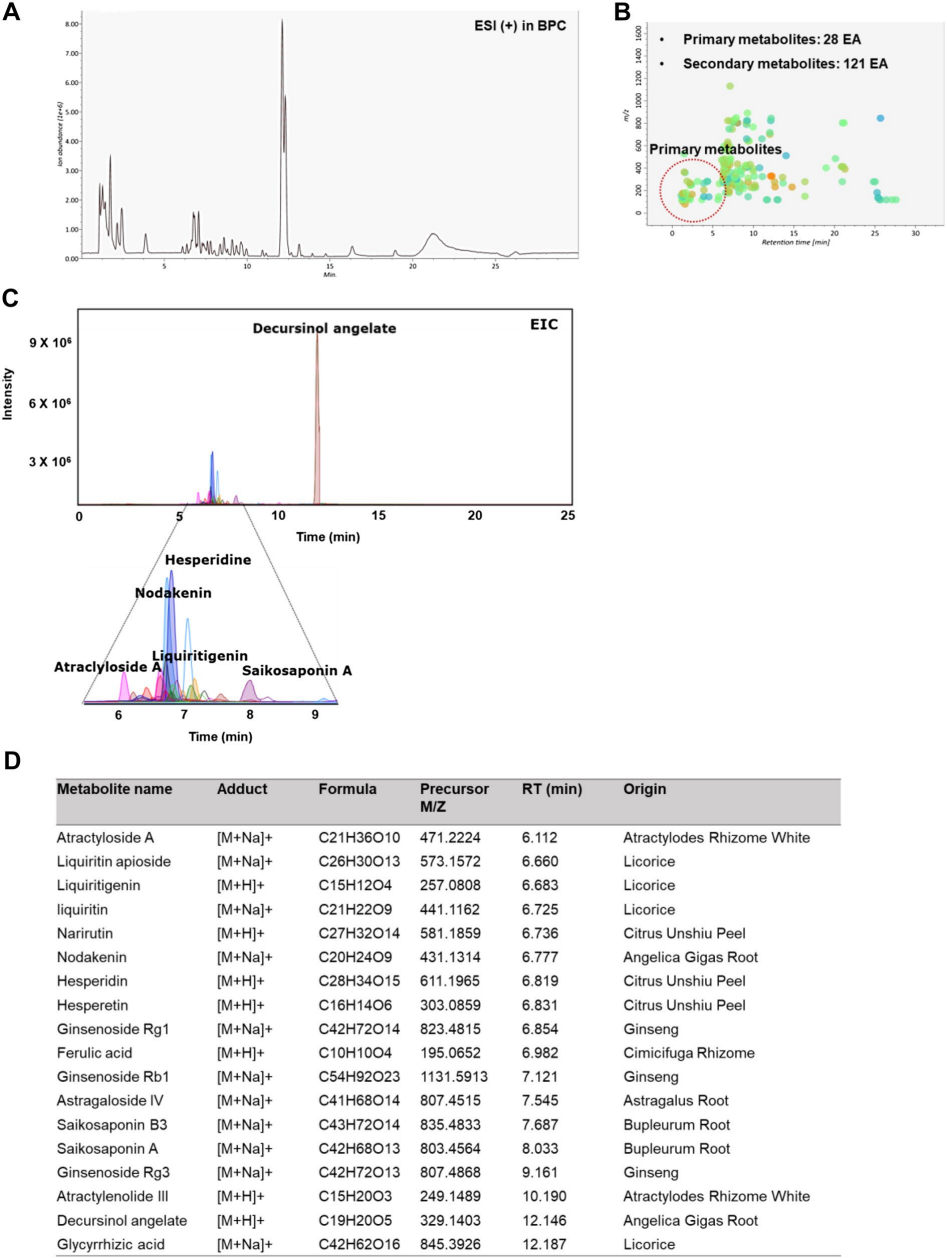
Chemical profiling of Bojungikgi-tang (BJIKT). **A** Base peak chromatograms (BPC); **B** A peak spot graph representing identified metabolites; **C** Extracted ion chromatograms (EIC) of six major constituents in BJIKT; **D** List of major chemical constituents in BJIKT

The major chemical constituents of BJIKT were determined based on UHPLC Q-TOF MS analysis according to their relative retention time, m/z of the precursor, and MS/MS fragments. A total of 1293 naked features were detected, and 149 metabolites were putatively matched with the reference peaks of the primary and secondary metabolites. We identified 18 major compounds, including astragaloside IV, atractylenolide III, atractyloside A, decursinol angelate, ferulic acid, ginsenoside Rb1, ginsenoside Rg1, ginsenoside Rg3, glycyrrhizic acid, hesperetin, hesperidin, liquiritigenin, liquiritin, apioside, narirutin, nodakenin, saikosaponin A, and saikosaponin B3, which were derived from each BJIKT extract. To standardize the BJIKT extract, we quantified the major components present in it, including decursinol angelate, hesperidin, nodakenin, liquiritigenin, atractyloside A, and saikosaponin A. The respective contents of these components were determined as follows: decursinol angelate (3.40 ± 0.00 mg/g), hesperidin (7.48 ± 0.01 mg/g), nodakenin (3.78 ± 0.12 mg/g), liquiritigenin (0.05 ± 0.04 mg/g), atractyloside A (0.06 ± 0.00 mg/g), and saikosaponin A (0.78 ± 0.02 mg/g) (Fig. 1).

### Effects of BJIKT on DNCB-induced AD symptoms in AD mice fed a low AhR ligand diet

**Fig. 2:**
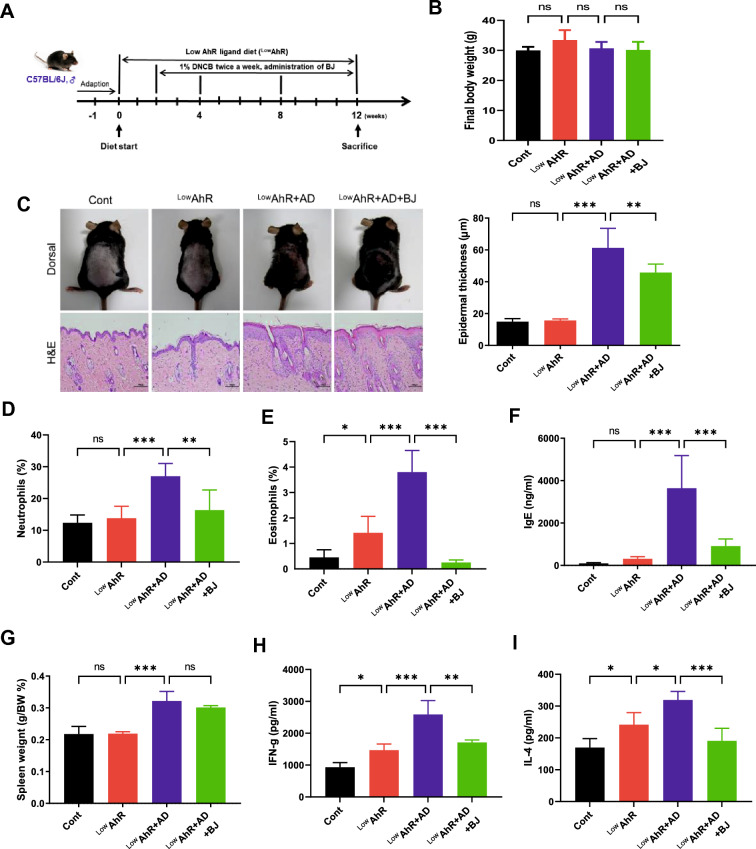
Effects of BJIKT on 2,4-dinitrochlorobenzene (DNCB)-induced atopic dermatitis (AD) symptoms in AD mice fed a low AhR ligand diet. **A** Timeline for AD mice fed a low AhR ligand diet and treatment with BJIKT; **B** Final body weight was scored 10 weeks after AD induction; **C** Evaluation of the total dorsal skin and epithelial thickness by histological analysis; measurement of the levels of **D** neutrophils and (**E**) eosinophils by CBC analysis; **F** IgE plasma levels; **G** spleen weight; and the levels of **H** interferon-gamma (IFN)-γ and **I** interleukin (IL)-4 in splenocytes. Number of experiments conducted for group comparisons: Cont, n = 6; ^Low^AhR, n = 6; ^Low^AhR + AD, n = 7; and ^Low^AhR + AD + BJ, n = 6. The values are expressed as the means ± standard error of the mean (SEM). *p < 0.05, **p < 0.01, ***p < 0.001 according to a one-way ANOVA followed by Tukey’s multiple comparisons

To investigate the effects of BJIKT on AD-like symptoms in AD mice fed a low AhR ligand diet, C57BL/6 mice were first sensitized with multiple applications of DNCB on their dorsal skin and then treated daily with BJIKT for 10 weeks (Fig. 2A). As a result of checking the final body weight, there was no significant difference between each group (Fig. 2B).

Repeated application of DNCB to mouse dorsal skin induced skin hypersensitivity reactions, such as erythema, hemorrhage, and scarring or dryness (Fig. 2C). Compared to the ^Low^AhR group, the ^Low^AhR + AD group (p < 0.001) showed significantly increased epidermal thickness due to epidermal keratinocyte hyperplasia, whereas the ^Low^AhR + AD + BJ group (p < 0.01) showed significantly decreased epidermal thickness compared to the ^Low^AhR + AD group **(**Fig. 2C**)**. Among the several subtypes of WBC, neutrophils (p < 0.001) and eosinophils (p < 0.001) showed that BJIKT suppressed the ^Low^AhR + AD-induced increases in the numbers of each cell type to similar extents (Fig. 2D, E).

Additionally, IgE levels were significantly higher in the ^Low^AhR + AD group (p < 0.001), whereas the ^Low^AhR group did not show statistical significance compared to the control group. However, IgE levels in the ^Low^AhR + AD + BJ group (p < 0.001) were considerably lower than those in the ^Low^AhR + AD group (Fig. 2F). Similarly, the substantially increased spleen weight in ^Low^AhR + AD mice (p < 0.001) slightly decreased in response to treatment with BJIKT treatment; however, the difference was not significant (Fig. 2G).

In the culture supernatant of splenocytes, the ^Low^AhR and ^Low^AhR + AD groups showed a considerable increase in IL-4 (p < 0.05, ^Low^AhR; p < 0.05, ^Low^AhR + AD) and IFN-γ (p < 0.05, ^Low^AhR; p < 0.001, ^Low^AhR + AD) levels compared to the control group (Fig. 2H, I). However, the ^Low^AhR + AD + BJ group showed a significant decrease in IL-4 (p < 0.001) and IFN-γ (p < 0.01) levels compared to the ^Low^AhR + AD group. These findings demonstrate that BJIKT attenuates AD-like symptoms in AD mice fed a low AhR ligand diet.

### Effects of BJIKT on skin barrier dysfunction in AD mice fed a low AhR ligand diet

**Fig. 3:**
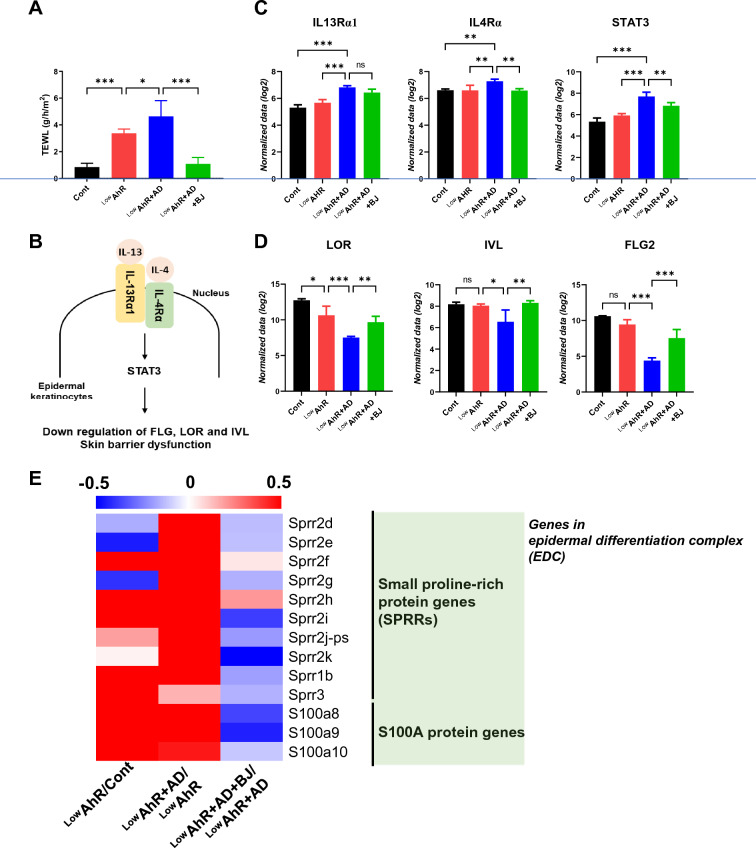
Effects of BJIKT on skin barrier dysfunction in AD mice fed a low AhR ligand diet. **A** Measurement of transepidermal water loss (TEWL) was performed at the end of the experiments after sensitization using TM300; RNA-seq analysis of the expression levels of the differentially expressed genes **B** Activation of the IL‐13/4‐STAT3 axis inhibits the expression of FLG and LOR (downregulation of barrier); **C** IL4Rα, IL13Rα1, STAT3 in the skin; **D** loricrin (LOR), involucrin (IVL), and filaggrin (FLG2) in the skin; **E** A representative heatmap of the RNA-seq analysis of skin tissues. Number of experiments conducted for group comparisons: Cont, n = 4; ^Low^AhR, n = 4; ^Low^AhR + AD, n = 4; and ^Low^AhR + AD + BJ, n = 4. The values are expressed as the means ± SEM. *p < 0.05, **p < 0.01, ***p < 0.001 according to a one-way ANOVA followed by Tukey’s multiple comparisons

To evaluate the effect of BJIKT on skin barrier dysfunction, we measured the TEWL. In general, TEWL is significantly increased and skin hydration is decreased in AD compared to that in healthy individuals [23]. In this study, TEWL values significantly increased in the ^Low^AhR (p < 0.001) and ^Low^AhR + AD (p < 0.05) groups compared to those in the control group. In contrast, administration of BJIKT (p < 0.001) significantly decreased the TEWL value compared to that in the control group (Fig. 3A).

The investigation employed RNA-sequence analysis of skin samples to delve into the underlying mechanisms pertaining to skin immune response, inflammation, and skin barrier function, as illustrated in Fig. 3B. The analysis revealed that in the ^Low^AhR + AD group, the IL-4Rα/IL-13Rα1 and STAT3 pathways were activated. Activation of these pathways can lead to the downregulation of key genes involved in skin barrier function, namely FLG (Filaggrin), LOR (Loricrin), and IVL (Involucrin). This downregulation of skin barrier genes can compromise the integrity of the skin barrier and potentially contribute to immune responses and skin inflammation [24]. However, administration of BJIKT appeared to inhibit the expression of IL-4Rα/IL-13Rα1 and STAT3 (Fig. 3C), indicating its potential to alleviate immune responses and skin inflammation.

Furthermore, a significant upregulation of epidermal barrier homeostasis genes (LOR, IVL, and FLG2) was observed following BJIKT treatment (p < 0.01, LOR; p < 0.01, IVL; p < 0.001, FLG2; Fig. 3D). These genes, which were downregulated in the ^Low^AhR and ^Low^AhR + AD groups, play critical roles in maintaining the skin's barrier function. Notably, many barrier-related molecules expressed in the granular layer are genetically mapped to the epidermal differentiation complex (EDC), including FLG, FLG2, LOR, and IVL. Additionally, the EDC encompasses clustered gene families such as the S100A protein genes (S100As) and small proline-rich protein genes (SPRRs). Significant upregulation of SPRRs and S100As was observed in the ^Low^AhR and ^Low^AhR + AD groups, and treatment with BJIKT suppressed these increasing expression trends **(**Fig. 3E**)**. These results indicate that BJIKT improves skin barrier dysfunction by regulating EDC gene expression in AD mice fed a low AhR ligand diet.

### BJIKT upregulates AhR target genes in AD mice fed a low AhR ligand diet

**Fig. 4:**
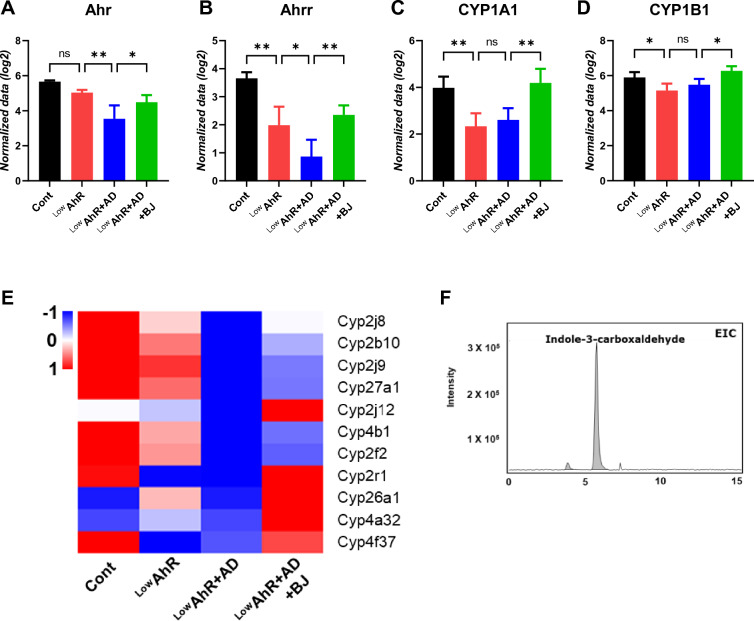
Upregulation of AhR target genes by BJIKT in AD mice fed a low AhR ligand diet. RNA-seq analysis of the relative expression of AhR target genes (**A**) Ahr, (**B**) Ahrr, (**C**) CYP1A1, and (**D**) CYP1B1 in the skin. (**E**) A representative heatmap of the differentially expressed Cytochrome P450 family genes in the skin. **F** TheeExtracted ion chromatograms (EIC) of indole-3-carboxaldehyde as an AhR ligand in BJIKT-treated cells. Number of experiments conducted for group comparisons: Cont, n = 4; ^Low^AhR, n = 4; ^Low^AhR + AD, n = 4; and ^Low^AhR + AD + BJ, n = 4. The values are expressed as mean ± SEM. *p < 0.05, **p < 0.01, according to a one-way ANOVA followed by Tukey’s multiple comparisons

We evaluated the expression of AhR target genes (Ahr, Ahrr, CYP1A1, and CYP1B1) to determine whether the effect of BJIKT on the skin barrier function of AD mice fed a low AhR ligand diet was mediated by the regulation of AhR signaling using RNA-seq analysis (Fig. 4A–D). Compared to the control group, AhR expression was significantly downregulated in the ^Low^AhR + AD group and decreased in the ^Low^AhR group. However, it was significantly upregulated in the ^Low^AhR + AD + BJ group. Moreover, the expressions of AhR target genes (Ahrr, CYP1A1, and CYP1B1) were downregulated in ^Low^AhR and ^Low^AhR + AD groups but were strongly upregulated in the ^Low^AhR + AD + BJ group. The heatmap of the RNA-seq data showed similar trends in the expression of the CYP gene family between the groups (Fig. 4E). The expression of CYP genes tended to be downregulated in the ^Low^AhR and ^Low^AhR + AD groups, whereas it was upregulated in the BJIKT-treated ^Low^AhR + AD group, but not significantly. Particularly, BJIKT more specifically regulates the CYP1 gene family in AhR signaling, rather than all CYP genes. In addition, we identified the AhR ligand indole-3-carboxaldehyde as the major constituent in BJIKT (Fig. 4F). These results indicate that BJIKT contains an AhR ligand and regulates AhR signaling by upregulating AhR target genes in AD.

### The effects of BJIKT in spleen immune cells in AD mice fed a low AhR ligand diet

**Fig. 5:**
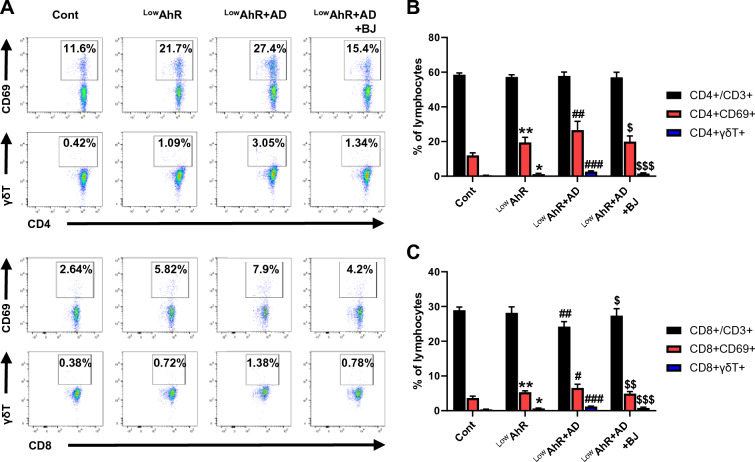
The effects of BJIKT on CD4 + and CD8 + T cell populations in AD mice fed a low AhR ligand diet. Fluorescence-activated cell sorting of CD4 and CD8 T cell populations in the spleen. **A** Gating strategy for identification of CD4 + /CD3 + , CD4 + /CD69 + , CD4 + /γδT + , CD8 + /CD3 + , CD8 + /CD69 + , and CD8 + /γδT + in splenocytes; percentage of **B** CD4 + T cells (CD4 + /CD3 + , CD4 + /CD69 + , CD4 + /γδT +) and **C** CD8 + T cells (CD8 + /CD3 + , CD8 + /CD69 + , CD8 + /γδT +) in splenocytes. Number of experiments conducted for group comparisons: Cont, n = 6; ^Low^AhR, n = 6; ^Low^AhR + AD, n = 7; and ^Low^AhR + AD + BJ, n = 6. The values are expressed as the means ± SEM. *p < 0.05, **p < 0.01 vs Cont; #p < 0.05, ##p < 0.01, ###p < 0.001 vs. ^Low^AhR; $p < 0.05, $$p < 0.01, $$$p < 0.001 vs. ^Low^AhR + AD. Only these two groups were compared and the significance was marked

We analyzed alteration in immune cell infiltration in the spleen using FACS (Fig. 5A–C). The absolute numbers of CD4 + /CD69 + and CD4 + /γδT + cells showed a significant increase in the ^Low^AhR and ^Low^AhR + AD groups, whereas the ^Low^AhR + AD + BJ group showed a significant decrease in the absolute number (CD4 + /CD69 + , p < 0.05 and CD4 + /γδT + , p < 0.001). However, there was no difference in the absolute number of CD4 + /CD3 + cells among groups. Similarly, the absolute numbers of CD8 + /CD69 + and CD8 + / γδT + significantly increased in the ^Low^AhR and ^Low^AhR + AD groups, whereas they were significantly decreased in the group receiving BJIKT group compared to those in the ^Low^AhR + AD group (CD8 + /CD69 + , p < 0.01; CD8 + /γδT + , p < 0.001). Compared to the ^Low^AhR group, the absolute number of CD8 + /CD3 + cells were significantly decreased in the ^Low^AhR + AD group and significantly increased by treatment with BJIKT (p < 0.05). These results suggest that γδT cells, despite being generally considered immunosuppressive, may exhibit unique functional properties that are augmented by a low AhR ligand diet and DNCB stimulation [25]. However, further experiments are needed to investigate the exact mechanisms underlying this observation.

**Fig. 6:**
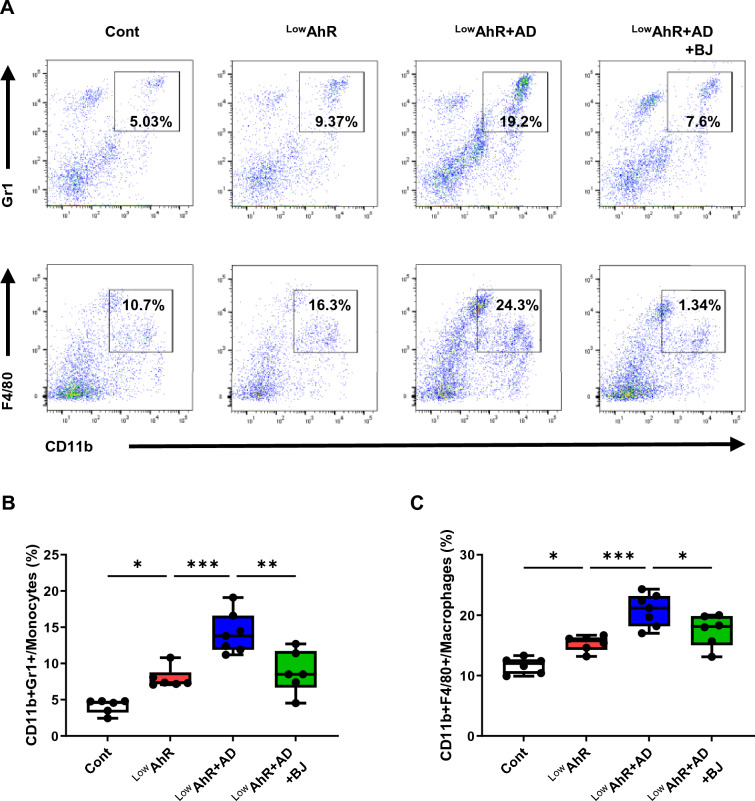
The effects of BJIKT on myeloid-derived suppressor cells (MDSCs) and macrophage cells in AD mice fed a low AhR ligand diet. Fluorescence-activated cell sorting of MDSCs (CD11b + Gr1 +) and macrophage (CD11b + F4/80 +) populations in the spleen. **A** Density plots showing gating strategies for MDSCs (CD11b + Gr1 +) and macrophage (CD11b + F4/80 +) cells; they show differences in percentages of **B** CD11b + Gr1 + and **C** CD11b + F4/80 + cells. Number of experiments conducted for group comparisons: Cont, n = 6; ^Low^AhR, n = 6; ^Low^AhR + AD, n = 7; and ^Low^AhR + AD + BJ, n = 6. The values are expressed as the means ± SEM. *p < 0.05, **p < 0.01, ***p < 0.001, according to a one-way ANOVA followed by Tukey’s multiple comparisons

The percentages of myeloid-derived suppressor cells (MDSCs; CD11b + Gr1 +) and macrophages (CD11b + F4/80) were significantly increased in the ^Low^AhR and ^Low^AhR + AD groups compared to the control group but significantly decreased in the ^Low^AhR + AD + BJ group compared to the ^Low^AhR + AD group (Fig. 6B, C). These findings indicate that BJIKT may also affect immune responses by suppressing several immune cell subtypes. We acknowledge the lack of significant difference in spleen weight despite changes in immune cell populations and propose possible explanations, including alterations in cell distribution, the balance of immune cell types, and the activation rather than proliferation of immune cells [26, 27], which should be further investigated.

### BJIKT-induced alterations in immune markers are correlated with the expression levels of skin barrier-related and AhR target genes

**Fig. 7:**
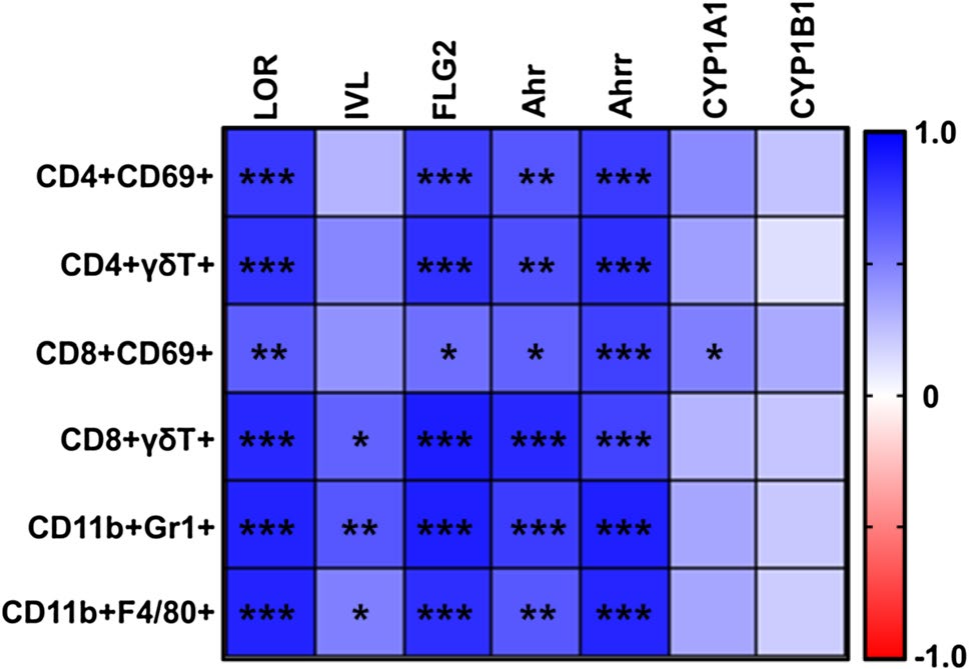
Correlation analysis between alterations in immune markers and skin barrier and AhR target gene expression levels after BJIKT administration. The heatmap shows the correlations between immune markers and barrier genes (LOR, IVL, and FLG2) and AhR target genes (Ahr, Ahrr, CYP1A1, and CYP1B1) levels in the skin. Pearson’s r values were determined using GraphPad Prism 9. The red color indicates a negative correlation. Correlation is significant at the *p < 0.05, **p < 0.01, ***p < 0.001 level

A correlation analysis was conducted to analyze the correlation between immune markers and skin barrier genes (LOR, IVL, and FLG2) and AhR target genes (Ahr, Ahrr, and CYP1A1). Figure 7 shows that the levels of immune markers associated with barrier genes (LOR, IVL, and FLG2) and AhR target genes (Ahr, Ahrr, and CYP1A1) were negatively correlated with immune cell subtypes. However, CYP1B1 expression was not significantly associated with immune marker expression. These results indicate that the immune response induced by BJIKT may alter the expression of the skin barrier and AhR target genes.

## Discussion

The role of AhR, a ligand-activated transcription factor involved in immune regulation and inflammation, is closely linked to chronic inflammatory skin diseases like AD and psoriasis [28]. We aimed to investigate the effects of the herb BJIKT in AD mice fed a diet low in AhR ligands, and to understand how AhR signaling influences AD development. It is important to note that our research has limitations, including the lack of direct comparisons between AD mice fed a normal diet and a low AhR ligand diet, as well as the absence of comparisons with positive control drugs or clinical drugs used for AD treatment. Future studies will aim to address these limitations to better evaluate the efficacy and limitations of BJIKT as a therapeutic agent for AD, thereby enhancing the interpretation and application of our findings in the field.

The results of the study demonstrated that BJIKT effectively improved AD symptoms by addressing multiple aspects of the condition. It reduced skin thickness, levels of blood neutrophils and eosinophils, IgE levels, and certain cytokines associated with immune response (IFN-γ and IL-4) in splenocytes. Additionally, BJIKT enhanced skin barrier function by reducing transepidermal water loss (TEWL) and modulating the expression of genes associated with skin barrier function and AhR signaling, particularly those related to the IL-4Rα/IL-13Rα1 and STAT3 pathways (Fig. 3C). These effects resulted in attenuated immune responses and inflammation [24].

Many protein products of epidermal differentiation encoded by EDC genes are differentially affected in AD and psoriasis via the AhR pathway [29]. So, we confirmed the expression of genes involved in the epidermal differentiation complex (EDC), such as SPRRs and S100As, in the skin. They found that these genes were significantly upregulated in the LowAhR and LowAhR + AD groups, but down-regulated in the BJIKT-treated LowAhR + AD group. Moreover, the study revealed that a low AhR ligand diet decreased the expression of genes in the canonical pathway of AhR signaling, including CYP1A1, CYP1B1, and Ahrr. However, BJIKT treatment was able to regulate the expression of AhR target genes in the canonical pathway and alleviate AD symptoms. These findings supported previous research emphasizing the role of AhR signaling in regulating genes involved in the EDC, which are crucial for skin barrier function and inflammation [30].

AhR plays a major role in several immune cell populations, such as lymphokine-activated killer cells, cytotoxic T cells, natural killer cells, B lymphocytes, T lymphocytes, and macrophages [31–34]. However, most studies have focused on immune modulation in the skin and intestine, and studies on the systemic immune system are rare. The study revealed that AD mice fed a low AhR ligand diet had higher levels of splenic immune cells in CD4 + and CD8 + T cells, macrophages, and MDSCs compared to the control group. These high immune cell levels were reduced by administration of BJIKT. Furthermore, the levels of immune cells negatively correlated with the expression levels of genes related to the skin barrier and AhR signaling, suggesting a connection between low AhR ligand diet, immune response aggravation, and AD. While we acknowledge the limitation of not directly assessing immune cells in the skin, we gained insights into the involvement of AhR signaling in the skin immune response by exploring the expression of AhR and its downstream signaling molecules in skin tissue through RNA-seq data.

The study also highlighted the importance of AhR ligands in maintaining skin barrier integrity. Previous studies have shown that coal tar, an AhR ligand, can repair the skin barrier in AD through regulation of the AhR pathway [35, 36]. Similarly, we found that BJIKT contains an AhR ligand called indole-3-carboxaldehyde, which exhibited protective roles against AD and exerted anti-inflammatory effects through AhR-CYP1A1 signaling in AD mice fed a low ligand diet.

However, the components of BJIKT, such as hesperidin [37], nodakenin [38], and liquiritigenin [39], have been shown to be effective against AD through mechanisms other than AhR signaling, such as suppressing Th17 activity or T cell activation. Therefore, the effect of BJIKT on AD may not be solely attributed to the regulation of the AhR signaling pathway. Further studies are needed to explore the combined effects of the AhR ligand and the components of BJIKT, as well as the previously reported effects of BJIKT components on the AhR signaling pathway, to gain a more precise understanding of the underlying mechanisms.

Nevertheless, the findings of this study highlight the molecular biological mechanism of AD under low AhR ligand diet conditions. The findings provided insights into the development of therapeutic agents for AD and laid a foundation for future research exploring the use of various AhR ligands in the treatment of this condition.

## Conclusions

The findings of this study present compelling evidence that BJIKT, as a treatment agent, demonstrated protective effects against AD induced by DNCB in mice fed a low AhR ligand diet. These results are particularly intriguing because BJIKT not only contributed to maintaining a healthy skin environment but also exerted its effects by modulating the immune response through alterations in the AhR pathway.

## Data Availability

The raw sequence and processed data were deposited in the NCBI Gene Expression Omnibus (GEO, https://www.ncbi.nlm.nih.gov/geo/ [accessed on 8 March 2023]) with accession number GSE226916. The data presented in this study are available on request from the corresponding author.

## Abbreviations

AhR: Aryl hydrocarbon receptor
AD: Atopic dermatitis
ANOVA: Analysis of variance
BJIKT: Bojungikgi-tang
CBC: Complete blood count
CYP1A1: Cytochrome P450 1A1
CYP1B1: Cytochrome P450 1B1
DNCB: 2,4-Dinitrochlorobenzene
EDC: Epidermal differentiation complex
ExDEGA: Excel-based differentially expressed gene analysis
FACS: Fluorescence-activated cell sorting
FICZ: 6-Formylindolo[3,2-b] carbazole
FLG: Filaggrin
H&E: Hematoxylin and eosin
IAld: Indole-3-aldehyde
IFN-γ: Interferon-gamma
IL: Interleukin
IVL: Involucrin
LOR: Loricrin
MDSCs: Myeloid-derived suppressor cells
SEM: Standard error of the mean
SPRRs: Small proline-rich protein genes
TCR: T-cell receptor
TEWL: Transepidermal water loss
